# Physical Activity after Cardiac EventS (PACES) – a group education programme with subsequent text-message support designed to increase physical activity in individuals with diagnosed coronary heart disease: study protocol for a randomised controlled trial

**DOI:** 10.1186/s13063-018-2923-x

**Published:** 2018-10-04

**Authors:** Louisa Y Herring, Helen Dallosso, Sudesna Chatterjee, Danielle Bodicoat, Sally Schreder, Kamlesh Khunti, Tom Yates, Sam Seidu, Ian Hudson, Melanie J Davies

**Affiliations:** 1 Leicester Diabetes Centre, University Hospitals of Leicester, Leicester General Hospital, Leicester, LE5 4PR UK; 2 Diabetes Research Centre, College of Medicine, Biological Sciences and Psychology, University of Leicester, Leicester, LE5 4PW UK; 3 NIHR Collaboration for Leadership in Applied Health Research and Care - East Midlands, University of Leicester, Leicester, UK; 4 NIHR Leicester Biomedical Research Centre, University of Leicester, Leicester, UK; 5 Department of Cardiology, Glenfield Hospital, Leicester, LE3 9QP UK

**Keywords:** Coronary heart disease, Physical activity, Education, Self-management, Randomised controlled trial, Cardiac rehabilitation

## Abstract

**Background:**

Coronary heart disease (CHD) represents approximately 13% of deaths worldwide and is the leading cause of death in the UK with considerable associated health care costs. After a CHD event, timely cardiac rehabilitation optimises patient outcomes. However, a high percentage of these services do not meet necessary performance indicators such as course length and follow-up attendance. Uptake of such services is only 50% in UK patients and support provided 12 months after an event is often limited. To delay and prevent further CHD events leading to hospitalisation, supplementary self-management strategies such as group education, are necessary.

**Methods:**

This is a single-centre, randomised controlled trial (RCT) recruiting participants (*n* = 290) aged ≥18 years who are 12 to 48 months post diagnosis of a CHD-related cardiac event (myocardial infarction, angina and any other acute coronary syndrome). The study aims to implement a structured education programme, with text-message support over 12 months, and identify whether delivery of the programme, to individuals who have a history of a cardiac event, would be an effective and cost-effective strategy for increasing walking. The primary outcome, objectively measured average daily physical activity, specifically step count through walking activity, is assessed using the wrist-worn GENEActiv accelerometer at baseline, 6 and 12 months. Secondary outcomes at 12 months include cardiovascular risk factors such as smoking status, blood pressure, lipid profile, glycated haemoglobin (HbA1c), obesity, self-efficacy, quality of life, physical activity and physical function.

Participants are randomised to either the control group receiving standard care and a physical activity information leaflet, or the intervention group whose partcipants receive the leaflet and are invited to attend two group-based structured education sessions. These encourage participants to adopt and maintain healthy behaviours and self-manage their lifestyle. They are delivered approximately 2 weeks apart by trained facilitators and reinforced via subsequent text-message support.

**Discussion:**

To our knowledge, this is the first trial designed to assess the effectiveness of a group education programme 12 to 48 months after a CHD event diagnosis. If successful, the PACES programme could be translated into effective post-operative cardiac care and complement the current post-operative services available.

**Trial registration:**

ISRCTN, ID: ISRCTN91163727. The trial was registered on 27 February 2017.

**Electronic supplementary material:**

The online version of this article (10.1186/s13063-018-2923-x) contains supplementary material, which is available to authorized users.

## Background

Cardiovascular disease (CVD) is the number-one cause of death globally representing 31% of all global deaths, with coronary heart disease (CHD) representing approximately 13% of deaths worldwide (7.4 million) [1]. CHD-related mortality in the UK accounts for one in six men and more than one in ten women [2]. Health care costs are considerable with nearly £2 billion per year spent on the treatment of CHD [3]. Whilst UK mortality rates from CHD have been decreasing since the 1970s, they are still relatively high compared to other countries in Western Europe [3, 4].

After a CHD event, the risk of further CHD events is greatly increased compared to the general population, unless there is intensive management of CHD risk factors including physical activity, smoking, diabetes, hypertension, hyperlipidaemia and obesity [5]. Following a CHD event, as per the UK National Service Framework, patients are offered a structured education and exercise programme of cardiac rehabilitation (CR) typically at 4 weeks after an acute cardiac event, after which risk factor management is usually transferred to primary care [6].

The UK National Audit of Cardiac Rehabilitation Annual Statistical Report 2016 [7] has compared current national figures with CR delivery recommendations. The current European guidance suggests that such services should be 12 weeks in duration and delivered soon after the cardiac event in order to promote long-term self-management [8]. Currently, the median duration of such programmes in the UK is 9 weeks [7]. At present only 50% of UK patients uptake CR multidisciplinary services [7]. Furthermore, even for those who have attended CR, support is limited thereafter [9]. To delay and prevent further CHD events leading to hospitalisation and potentially death, supplementary strategies are necessary.

### Physical activity in the prevention of subsequent CHD events

Performing physical activity regularly is independently associated with a decrease in CHD risk, which elicits a positive dose-related response on cardiovascular risk factors and should be a predominant focus in both primary and secondary care [10]. Individuals with CHD benefit significantly from physical activity, with higher fitness levels predicting lower mortality rates and CHD-associated complications [11]. The large multi-centre NAVIGATOR trial demonstrated that subsequent risk of a cardiovascular event is inversely associated with both baseline levels and change in ambulatory activity in individuals at high CVD risk [12]. Specifically, an increase or decrease in daily ambulatory activity of 2000 steps between baseline and 12 months was associated with an 8% higher or lower CVD event risk, respectively [12]. These data strongly support interventions that increase physical activity in high-CVD-risk patients.

Previous UK studies have shown benefits in a number of physical activity parameters through structured education and physical activity [13, 14]. Let’s Prevent, a large NIHR-funded randomised controlled trial (RCT), extensively evaluated the implementation of a structured education programme in people at increased risk of developing type 2 diabetes and showed improvements in step count and sedentary time [14]. The MOTION study, which looked at effects of structured exercise in non-diabetic subjects 12–24 months after bariatric surgery, has shown benefits in improved physical activity, weight and functional performance; specifically, the Incremental Shuttle Walk Test (ISWT) [13]. The ISWT is a valid field test of fitness in patients undergoing conventional CR. Following cardiac rehabilitation 62% of patients achieve the minimum clinical difference of > 70 m [7].

After completing CR, patients are encouraged to self-manage their condition and to pursue a healthy lifestyle. Patients’ long-term management plan should be a collaboration between the patient and primary and secondary care services [8]. The National Service Framework for CHD (2000) states that 1 year after cardiac rehabilitation, at least 50% of patients should be undertaking 30 min of moderate physical activity at least five times a week, maintaining a Body Mass Index (BMI) of less than 30 kg/m^2^, and not smoking. UK National Audit figures for 2011–2012 showed that 12 months after cardiac rehabilitation there was a 14% increase in exercise levels, a 1% reduction in BMI in patients with a BMI < 30 kg/m^2^, and a reduction in smoking of 4% [15]. Physical inactivity accounts for 76% of England’s cardiac rehabilitation population upon referral to cardiac rehabilitation [16], and there is a 30% increase in individuals meeting the physical activity guidelines of 150 min per week after completing cardiac rehabilitation [7]. It is well noted that timely cardiac rehabilitation optimises patient outcomes; however, patient support is limited after discharge back to primary care [9].

### Lifestyle education and remote text-message support

Research suggests that inadequate support is available after the initial 12-month period post cardiac event, with half of patients not taking up post-operative support offered. Regular face-to-face contact with health care professionals is expensive and time-consuming and cost-effective strategies to deliver interventions are urgently required in the UK. The delivery approach of behaviour change and self-management education for chronic diseases, such as CHD, should be directly relevant to primary care pathways [17]. It is recognised that structured education is a method of promoting self-management in clinical populations, specifically those with CHD, and that ambulatory physical activity positively affects future cardiovascular event risk [18].

With the advent of increasingly sophisticated mobile phone technology, text messaging has been used to deliver health education messages which reinforce and promote behavioural change. Whilst some studies suggest that text-message reminders of health education and medication are not beneficial in terms of self-efficacy [19], others have demonstrated that it can be used effectively to reduce CHD risk; for example, by preventing type 2 diabetes [20], increasing levels of leisure-time physical activity and walking [21], or adherence to antiplatelet therapy [22]. A recent mixed-methods observational cohort study of automated text messaging and remote nursing as part of mobile phone diabetes programmes demonstrated improvements in the taking of medications, healthy eating, foot care, exercise, and glucose monitoring as well as self-efficacy, social support, and health belief measures [23]. In this study, behavioural theory was used to inform, identify and evaluate efficacy of the mobile phone intervention.

It is apparent, given the above factors, that more support 1 year after a cardiac event is needed to complement current services. There is a need to develop and evaluate a structured education programme focusing on lifestyle factors, such as physical activity, for individuals who have a history of a cardiac event.

## Methods

### Aims and objectives

1. To implement an acceptable and effective structured education programme with text-message support for increasing total daily physical activity, specifically walking activity measured using accelerometry, and reducing subsequent cardiovascular events in individuals 12 to 48 months after diagnosis of a cardiac event
2. To assess the effectiveness of a structured education intervention to improve cardiovascular risk factors such as smoking, blood pressure, lipid profile, obesity, self-reported physical activity and objectively measured physical activity intensity
3. To assess the acceptability, uptake and feasibility of implementing the programme in a population at high future risk of another CVD event in primary care

### Study overview

**Fig. 1:**
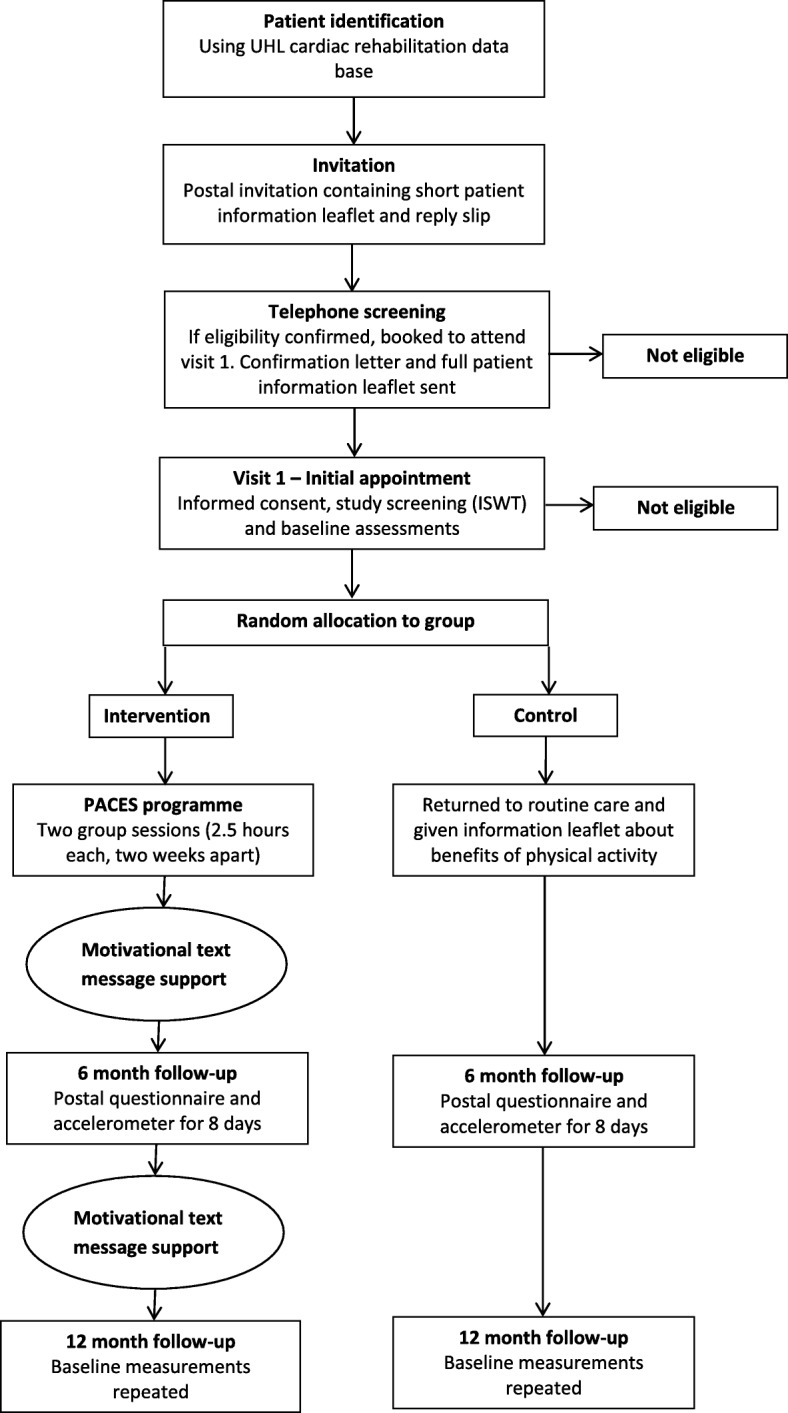
Flowchart of research procedures

**Fig. 2:**
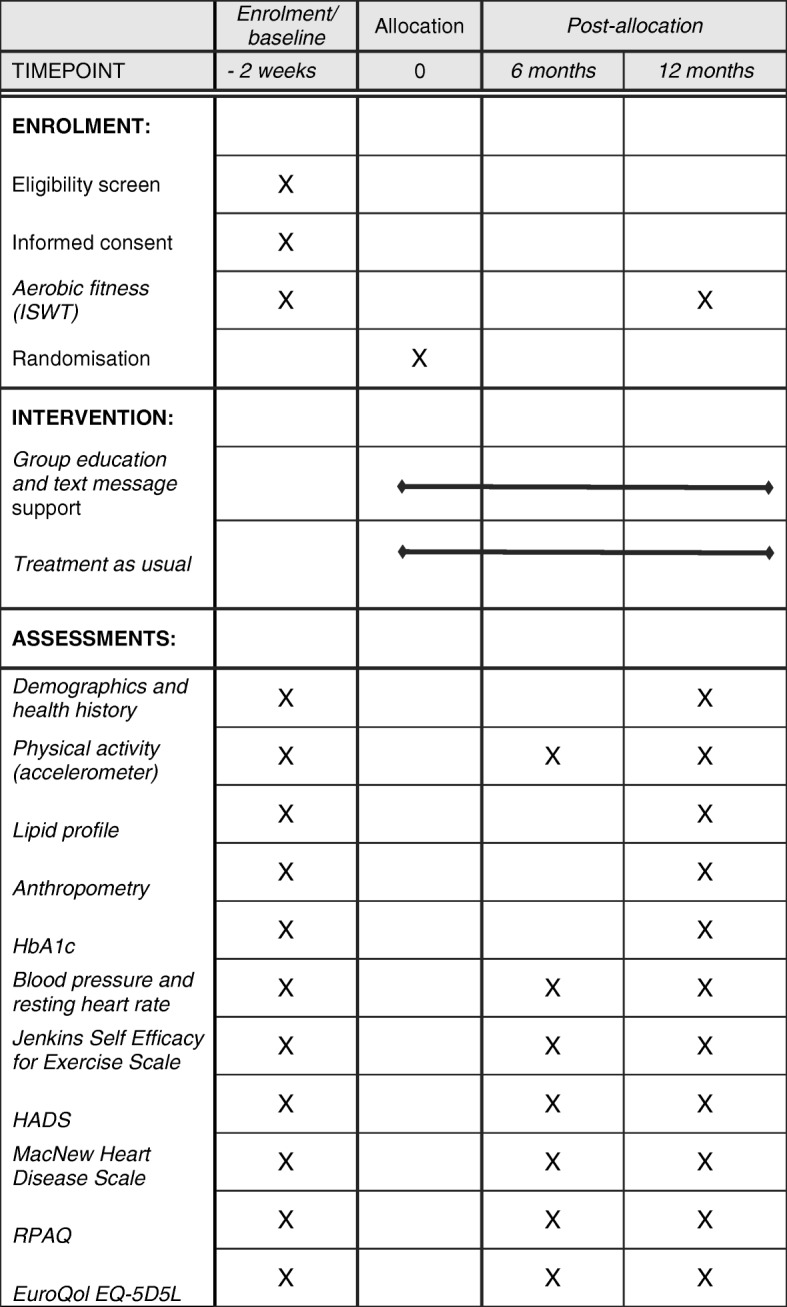
Standard Protocol Items: Recommendations for Interventional Trials (SPIRIT) diagram. Abbreviations: *ISWT* Incremental Shuttle Walk Test, *HbA1c* glycated haemoglobin, *HADS* Hospital Anxiety and Depression Scale, *RPAQ* Recent Physical Activity Questionnaire

This single-centred, two-arm, parallel, 12-month RCT co-ordinated from the University Hospitals of Leicester NHS Trust compares structured disease management education, delivered as a group education programme followed by text-message support, with usual care. The Physical Activity after Cardiac EventS (PACES) education programme has been designed according to the Medical Research Council guidelines for developing and evaluating complex interventions in health behaviour change programmes, along with Michie et al*.* and the NICE guidelines, to inform behaviour-change techniques [24–26]. The main goal of the PACES programme is to increase physical activity; specifically, walking activity. Figure 1 describes the study flow and participant progression through the PACES study. The trial is sponsored by the University of Leicester and approval was granted by the West Midlands – Solihull Research Research Ethics Committee and the UK Health Research Authority. The study was prospectively registered (ISRCTN91163727). The protocol is reported according to the Standard Protocol Items: Recommendations for Interventional Trials (SPIRIT) guidelines; see Fig. 2 and Additional file 1.

### Participant invitation and recruitment

Participants are identified 12–48 months after a CHD cardiac event diagnosis from the University Hospitals of Leicester cardiology department and phase 4 community-based cardiac rehabilitation providers. Phase 4 cardiac rehabilitation typically refers to community-based cardiac rehabilitation, which provides people with known coronary heart diseases (myocardial infarction, coronary artery bypass graft, percutaneous coronary intervention and stable angina), the possibility to benefit from weekly supervised exercise sessions. People who are eligible for phase 4 cardiac rehabilitation include those who have participated in a 6–12-week cardiac rehabilitation programme. Phase 4 is often referred to as a maintenance programme and is only available to those who meet certain medical criteria and have been assessed by their general practitioner (GP).

Potential participants identified as living in Leicestershire and within the post-CHD event diagnosis window (12–48 months) are invited to participate by postal invitation. The postal invitation includes an invitation letter and a short version of the participant information leaflet. A follow-up telephone call is made to non-responders to check that they have received the invitation and ask if they have any questions relating to the PACES study and its procedures. All positive responders to the postal invitation receive telephone screening related to the inclusion and exclusion criteria and are provided with an opportunity to ask questions related to the study. All potential participants who pass the telephone screening phase are invited to visit 1 to ensure that they meet the practical elements of the inclusion and exclusion criteria. If deemed able and eligible to participate, written informed consent is obtained after which baseline assessment measurements are carried out (Table 1) and randomisation takes place. Ahead of visit 1, the potential participant receives an appointment confirmation letter and the PACES full participant information leaflet. These visits take place at University Hospitals of Leicester premises or in community hospitals where appropriate resuscitation facilities are available.

**Table 1:** Screening, primary and secondary outcome measures

	Measurement type	Measurement (units)	Baseline	6 months	12 months
Screening	Demographic data	Date of birth, age, gender, ethnicity,	x		
		smoking status, alcohol status employment status	x		x
	Health history	Type of cardiac event defining as eligible, date of CHD diagnosis,	X		
		medical history and medications, whether family history of CHD	x		x
Primary outcome measure	Physical activity	Accelerometer (average daily physical activity - milli-gravitational units (mg))	x	x	x
Secondary outcome measures	Aerobic fitness	Incremental Shuttle Walk Test (metres)	x		x
	Anthropometric	Height (cm) and body mass (kg)	x		x
		Body Mass Index (kg/m^2^)			
		Waist circumference (cm)			
		Hip circumference (cm)			
		Waist to hip ratio			
	Cardiovascular	Blood pressure (mmHg) and resting heart rate (bpm)	x		x
	Blood samples (all non-fasting)	Lipid profile (cholesterol, HDL, LDL, triglycerides)	x		x
		HbA1c (mmol/mol, %)			
	Questionnaires	Jenkins Self-efficacy for Exercise Expectations Scale [45]	x	x	x
		Hospital Anxiety and Depression Scale (HADS) [58]	x	x	x
		MacNew Heart Disease [43, 44]	x	x	x
		Recent Physical Activity Questionnaire (RPAQ) [40]	x	x	x
		EuroQoL (EQ-5D-5L) health-related quality of life instrument [43, 44]	x	x	x
		Morningness-Eveningness Questionnaire [49, 50]	x		
Exploratory endpoint	Stored serum and plasma samples	Biomarkers of inflammation, proteomics, metabolomics and novel markers of cardiovascular health	x		x

*CHD* coronary heart disease, *HbA1c* glycosylated hemoglobin, *HDL* high-density lipoprotein, *LDL* low-density lipoprotein

### Eligibility criteria

To be eligible for the PACES study individuals must meet the following criteria:

- Aged 18 years or older
- Be 12–48 months post confirmed diagnosis of a cardiac event (myocardial infarction, angina or acute coronary syndrome)
- Able to speak and read English in order to participate effectively in a group education programme
- Willing and able to attend the education sessions and clinic visits
- Willing and able to give informed consent
- Access to a mobile phone in order to receive text messages
- Willing to allow their GP notification of their participation in the study and access to patient records for purpose of the study
- Able to take part in moderate physical activity as assessed using the ISWT (level 3 or above (120 m)) [27]

Individuals will be excluded from the PACES study if they have/are:

- A diagnosis of heart failure where the underlying primary cause is not myocardial disease as a result of atherosclerosis
- Musculoskeletal limitations that limit physical activity (e.g. musculoskeletal injury)
- Participating in another clinical intervention study or have done so in the past 12 weeks
- A severe life-threatening co-morbidity (e.g. malignancy)
- Poor exercise capacity, (< level 3 on the ISWT (120 m)) [27]
- Housebound or immobile
- Unstable symptoms (chest pain or breathlessness at rest; unstable stage II hypertension (160/100 mmHg), not on necessary medications)

### Algorithm for exclusion of individuals with poor exercise capacity and unstable symptoms

All individuals who display a potential risk if they were to increase their physical activity levels are screened out using the unstable symptom check or ISWT and referred to the necessary support networks in primary or secondary care. This is to ensure that complications or symptoms they are experiencing are addressed.

### Randomisation

Upon completion of visit 1, participants are randomised using a 1:1 block design and stratified by gender (men; women) and ethnicity (White European; other) to receive either standard management in primary care or to a complex intervention with group-based structured education and text-message support. An exception is made for people living in the same household, where in the unlikely event of this occurring the second person is not randomised but is allocated to the same arm as the first person. This is to prevent contamination between people living in the same household but randomised to different arms. The randomisation schedule and list was developed by an independent statistician and allocation of randomisation is carried out by a researcher independent of the team. Participants are informed of their randomisation allocation once visit 1 is completed, ahead of their 6 and 12 months’ follow-ups. As the intervention is a group education programme the participants cannot be blinded to the randomisation. The research nurses collecting the follow-up data are not informed of the randomisation of the participant but it is possible that the information is passed onto them by the participant.

### Treatment regimens

#### Control group

Control group participants are provided with general health advice in the form of a standard British Heart Foundation information leaflet after visit 1, and are returned to standard care delivered by their GP. The information leaflet entitled ‘*Put Your Heart into Walking*’ describes walking as a fitness activity to keep your heart healthy [28].

#### Intervention group

Participants randomised to the intervention group receive the standard information leaflet after visit 1 and are invited to attend the PACES education programme which comprises two group-based structured education sessions.

Development of the intervention followed an iterative pathway comprising various stages of designing, testing and refining the education programme through the use of informant groups [29]. The theoretical underpinnings employed and processes used to inform the behaviour-change techniques in the programme include those recommended by Michie et al*.* [26], the NICE 2014 guidelines [25] and the behaviour-change taxonomy [26]. The COM-B system has been employed as an overarching model for understanding the factors underlying behaviour and behavioural change; namely, capability, motivation and opportunity [30]. This structured approach ensures that all aspects of the programme have a rationale and an aim.

The importance of tailoring interventions to meet the needs of the targeted clinical population has been recognised. All development stages, therefore, comprised of extensive patient and public involvement (PPI) work with health care professionals based in primary care and cardiac rehabilitation and with cardiac patients. Both groups worked closely with the development team to create a bespoke group-based education programme. The education sessions are 2.5 h in duration and delivered by two trained facilitators approximately 2 weeks apart. Sessions are delivered in a facilitative style that encourages participation with the use of reflective questioning and problem-solving activities to promote engagement and build self-efficacy. The PACES programme content is underpinned by an integrated theoretical framework encouraging the adoption and maintenance of healthy behaviours and lifestyle; specifically, increasing physical activity levels through daily walking activity, along with diet. The following topics are covered:

- What is CHD?
- Risks and how to reduce these risks to maintain a healthy heart
- The benefits of physical activity on CHD
- The importance of medication and healthy eating
- Improving confidence and personal motivation to increase physical activity
- Recognition of the personal barriers and facilitators to physical activity
- How to assess and goal plan physical activity using a self-monitoring tool (the participant is given a pedometer and activity diary to help with self-monitoring)

All attendees subsequently receive physical activity-related motivational reinforcement via text-message support. The participants receive 82 physical-activity-related text messages at different weekly frequencies following the second education session until the 12-month follow-up assessment. The text messages are non-interactive and are designed to be motivational in nature. The text-message support used is a validated package of messages designed and shown to be effective in the prevention of recurrent cardiovascular events [31, 32]. The current study is a pragmatic trial, and the target is to attend the education within 2 months of recruitment. However, this will not always be possible. A sensitivity analysis will be performed to take this into account.

##### Facilitator recruitment and training

Potential facilitators initially responded to an advertisement or attended a recruitment event after which they underwent shortlisting according to certain criteria necessary to the position (a registered health care professional or a professional in the field of health, health promotion or sports science, knowledge of CHD, experience working face-to-face with the general public and experience of group working).

All recruited facilitators attended an initial 2-day training course (session 1 general facilitator training and session 2 study-specific training, 15 h training in total) to ensure that they understood the theories and philosophy that underpin the PACES programme. The training course also covered the PACES curriculum content and ensured familiarisation with the teaching resources used within it. All facilitators were provided with a curriculum and resource pack to support a successful delivery and give them the opportunity to plan ahead of each session. The facilitators then delivered a ‘have a go session’ before the study started to enable them to practise delivery to a volunteer cardiac patient group. These practice sessions were observed by a member of the training team and feedback and further training were provided to the facilitators as necessary. Continued peer support is provided to the facilitators and self-reflection and peer reflection after every session is encouraged.

##### Intervention fidelity

An intervention fidelity tool has been developed to monitor and report adherence in a predetermined sample of the programmes delivered. It includes an ‘adherence measure’ to capture delivery (mode of delivery/duration/content) and use of resources (materials/activities) and a structured observation tool to assess facilitator delivery of prescribed behaviours and behaviour-change techniques [33]. The observation tool also includes an assessment of ‘talk time’ as a measure of quality of delivery [34]. Observation of delivery is being undertaken by trained observers who have been assessed as reliable in the use of the structured observation tool [33].

### Study outcomes

All clinical visits are run by trained individuals, predominantly research nurses and health care assistants from the University Hospitals of Leicester. All research staff have been trained in the study procedures and follow standard operating procedures (SOPs) when doing so. Written informed consent is obtained by a trained research nurse before any trial activities take place. Outcome data are collected at three time points – baseline, 6 months (postal) and 12 months (Table 1). Participants and their GPs are sent a letter with details of selected clinical results after the baseline and 12-month clinic visits.

The following demographic and medical history data are recorded for each participant: date of birth, gender, ethnic background, family history, employment status, smoking status, alcohol status and details of any relevant history of disease, medications, or relevant surgical interventions.

### Primary outcome

The primary outcome measure is change in objectively measured ambulatory activity from baseline to 12 months using the GENEActiv wrist-worn, tri-axial accelerometer (GENEActiv model 1.1, ActivInsights Ltd., Kimbolton, UK) with a dynamic range of ± 8 g, where g is equal to the earth’s gravitational pull. Increasing physical activity is an important behaviour to adopt after a cardiac event illustrating why objective ambulatory activity has been selected as the primary outcome. Participants are asked to wear the GENEActiv accelerometer on their non-dominant wrist for eight consecutive days (24 h) (wearing the monitor from the date of the assessment visit or from a specified date when sent in the 6-month postal follow-up). The accelerometer is configured to collect data at 100 Hz and records total physical activity, which includes light, moderate, vigorous and moderate to vigorous physical activity (MVPA), sedentary behaviour and sleep in milli-gravitational units (mg). Participants are also asked to complete a log whilst wearing the monitor to provide their waking hours and wear-time information. An appropriately trained individual instructs the participant on correct placement of the monitor using the SOPs. Participants are given a stamped addressed envelope to return the monitor once completed. Upon return the data are downloaded to a computer using the software supplied by the manufacturer and are then ready for analysis. The accelerometers are processed by staff blinded to the intervention group allocation.

### Secondary outcomes

#### Anthropometric measures

Body mass is measured using the bioelectrical impedance Tanita Scales BC-418-MA (Tanita Corporation, Tokyo, Japan) and stretch stature is measured using a portable stadiometer (Holtain, Crymych, UK). Body mass (kg) and stretch stature (cm) values are used to calculate Body Mass Index (BMI) as body mass (kg) divided by height (m) squared. Waist (approximately 1 cm above the iliac crest) and hip (widest area around the gluteus maximus) circumferences are recorded and waist:hip ratio (WHR) calculated. These measurements are included as an indicator of abdominal obesity [35].

#### Cardiovascular measures

Blood pressure and resting heart rate are obtained after participants have been seated for at least 5 min using the Omron HEM-907 Digital Upper Arm Cuff Blood Pressure Monitor (Omron Corporation, Kyoto, Japan). Participants remain seated with their left arm supported whilst the measurement is taken. Blood pressure is taken three times; the first measurement is discarded and a mean of the following two measurements is reported.

#### Functional walking measurement

The Incremental Shuttle Walk Test (ISWT) is used as a screening measure, as well as a secondary outcome measure, as it reflects walking ability, an important measure of daily living in these clinical patients. The ISWT has been validated against VO_2_ max and VO_2_ peak in clinical populations [36, 37]. A linear relationship is reported between functional capacity and the number of shuttles completed in a clinical population [37].

The ISWT involves a patient walking consecutive 10-m shuttles in time with an audible beep that becomes progressively faster, until they are no longer able to maintain that pace. The test has a total of 12 levels lasting 1 min each (total distance 1020 m). Participants perform a practice ISWT to minimise the influence of learning effects and are then asked to walk for as long as possible until reaching test termination criteria whilst the assessor records the total number of shuttles performed [38, 39]. The participant remains in the clinical area for at least 15 min following the test where measures of blood pressure, heart rate, oxygen saturation, rating of perceived exertion (RPE Borg Scale) and breathlessness (the modified Borg Dyspnoea Scale) are taken. The participant must achieve level 3 or above on the ISWT (120 m), and, if they are not able to achieve this, they are excluded from the study and directed back into cardiac rehabilitation (a letter is sent to the cardiac rehabilitation team suggesting referral; their GP is sent a copy of this letter) as a process of good practice [27]. This test is conducted by a basic life support (BLS)- or immediate life support (ILS)-trained member of the study team, experienced in conducting the ISWT. If the ISWT is being conducted by a BLS-trained individual, an ILS-trained member of staff is also available within the PACES clinic.

#### Questionnaire data

##### Recent Physical Activity Questionnaire (RPAQ)

The RPAQ is designed to explore day-to-day physical activity levels in the past 4 weeks. The questionnaire is divided into three sections: (1) physical activity patterns in and around the house, (2) travel to work and work activities and (3) recreational activities. This questionnaire has been validated against previous studies and is comparable when estimating energy expenditure and MVPA [40, 41].

##### Hospital Anxiety and Depression Scale (HADS)

The HADS is a validated scale measuring the severity of symptoms of anxiety and depression. It comprises 14 statements, of which seven relate to anxiety and seven relate to depression [42]. Each statement has an option of four responses scored from 0 to 3. Upon completion, selected scores are totalled and reported for anxiety and depression individually [42].

##### EuroQoL EQ-5D-5L

The EQ-5D assesses health-related quality of life and provides useful data for health economic analyses. The EQ-5D-5L is a validated measure of health status and has been validated specifically in chronic conditions, such as cardiovascular disease. The EQ-5D-5L has five quality of life dimensions (mobility, self-care, usual activities, pain/discomfort and anxiety/depression) which are all coded between 1 and 5 [43, 44].

##### Jenkins Self-efficacy for Exercise Expectations Scale

This validated self-efficacy scale measures ability to continue exercising in the face of nine barriers to exercise [45]. These barriers are specifically relating to when you are bothered by the weather, boredom, pain, exercising alone, lack of enjoyment, busyness, tiredness, stress and depression.

##### MacNew Heart Disease health-related quality of life instrument

The MacNew Heart Disease health-related quality of life instrument is a validated questionnaire designed to appraise how CHD affects emotional, physical and social functioning along with daily activity [46]. The MacNew is a 27-item questionnaire which is divided into the three factors of social functioning, physical functioning and emotional functioning [47, 48].

##### Morningness-Eveningness Questionnaire

The Morningness-Eveningness questionnaire is a 19-item self-assessment questionnaire that determines morningness-eveningness in human circadian rhythms (chronotype) [49, 50]. Disruption of circadian rhythms is reported as a significant risk factor for many cardiovascular diseases [51].

#### Laboratory tests – blood samples

Venous blood samples are taken during the baseline and follow-up clinics and sent for analysis of full lipid profile and HbA1c in accredited laboratories at University Hospitals of Leicester. The samples are analysed in accordance with the laboratory’s SOPs. All laboratory results are reviewed and the reports signed by the study medic who records in the case report form (CRF) whether they are normal, abnormal but not clinically significant, or abnormal. If abnormal, a letter is sent to the participant’s GP.

##### Exploratory endpoint

The feasibility for the measurement of biomarkers of inflammation, proteomics, metabolomics and novel markers of cardiovascular health will be determined as new knowledge and methods for assaying evolve. When providing informed consent, participants have the option to consent to the storage of their samples. If consent is obtained, additional samples are obtained, centrifuged at the point of collection, and stored as plasma and serum samples in a − 80 °C freezer using standardised stable methodology within the Leicester Diabetes Centre.

### Sample size

The PACES study power calculation was based on the primary outcome of change from baseline to 12 months of average daily physical activity, as quantified by the Euclidean norm minus one (ENMO) method measured in milli-gravitational units (mg). This is the main measure of activity derived from the GENEActiv activity monitor. In order to detect a minimum clinically significant difference of 2.1 mg, which is equivalent to an overall increase in physical activity volume of approximately 30 min of light walking at 4 km/h, assuming a standard deviation (SD) of 5.3 mg [52], a power of 80% and significance level of 5%, the sample size requires 202 participants. To allow for 20% loss to follow-up and 10% non-compliance of the activity monitor, 290 participants will be recruited to this trial (145 in each group).

### Data analysis

A Consolidated Standards of Reporting Trials (CONSORT) study flowchart will detail the movement of participants throughout the PACES study [52]. Baseline descriptive characteristics will be summarised by treatment arm. Continuous variables will be expressed as mean values (and SDs), or median values (with lower and upper quartiles) where appropriate. Binary and categorical variables will be expressed as number (percentage). Data will be checked for parametric assumptions. A complete case population (i.e. those with complete data for the primary outcome) will be analysed as the primary analysis.

#### Primary outcome data processing and data analysis

The primary outcome, change in average daily physical activity measured by the GENEActiv in mg from baseline to 12 months will be processed ahead of analysis. The GENEActiv data are downloaded using GENEActiv software version 3.1 and stored as raw bin files by assessment time point. These files will be analysed using the most up to date R-package, GGIR, using the version that is most up to date when analysis happens [53, 54]. Automatic calibration using local gravity as a reference, recognition of sustained abnormally high values, calculation of the average resultant vector magnitude, corrected for gravity and expressed as ENMO in mg averaged over 1-s epochs [54]. Files will be excluded from analyses if post-calibration error was greater than 0.01 g or fewer than 4 days of 16 h of wear-time were recorded by the monitor. Non-wear is estimated based on the SD and value range of each axis, calculated for 60-min windows with 15-min moving increments. If for at least two out of the three axes the SD is less than 13 mg or the value range is less than 50 mg the time window is classified as non-wear. Output variables will include overall physical activity and time spent inactive, in light physical activity and in moderate-to-vigorous physical activity.

After processing the primary outcome data the treatment arms will be compared using linear regression modelling with (1) a binary indicator for randomisation group as the explanatory variable, (2) terms for the stratification factors as confounders and (3) adjustment for the change from baseline in accelerometer wear-time and baseline average daily physical activity. Apart from those living in the same household, individuals are stratified by gender (men; women) and ethnicity (White European; other). Sensitivity analyses will include a per-protocol analysis, an intention-to-treat analysis where missing data are imputed using multiple imputation or another suitable method, and analyses with the intervention arm restricted to participants who attend the education within 2 months of recruitment. Interaction effects will be fitted between intervention arm and gender (male vs. female), and ethnicity (White European vs. other). If the interaction term is statistically significant at the 10% level then stratified analyses will be performed for that factor using the same model as the primary analyses.

Secondary outcomes will be analysed using similar methods as the main analysis, with an appropriate model selected dependent on the distribution of the outcome. The results of all comparative analyses will be presented with 95% confidence intervals and statistical significance for main effects will be assessed at the 5% level. All *p* values shown will be two-sided. Statistical significance for interaction effects will be assessed at the 10% level. Any deviation(s) from the original statistical analysis plan will be described and justified in the final report.

### Data management and monitoring

Data are entered on a validated electronic-password-protected data base on a University of Leicester server, with only the participant ID number included. Hard copies of the data are stored in locked filing cabinets and will be destroyed 10 years after the end of the study. The study is being conducted in accordance with the Research Governance Framework for Health and Social Care, ICH GCP and the Data Protection Act.

As this is a minimal risk study a Data Safety Monitoring Committee has not been convened. All staff working on the study have completed the required Good Clinical Practice training and follow the sponsor’s SOPs throughout the study. Serious adverse events (SAE) are monitored and reported in line with requirements. The study will participate in an external audit if requested by the sponsor. An internal group meets every month to review recruitment rate, drop out, issues concerning delivery of the intervention and SAE. A quarterly report on progress is submitted to the funder.

## Discussion

To the best of our knowledge, this is the first trial to deliver a lifestyle intervention combined with follow-up text-message support in this population 12–48 months after diagnosis of a CHD cardiac event. It is well noted that lifestyle education and intensive risk factor management is needed after a CHD event to minimise the increased risk of future events occurring [5, 6]. Given that a high percentage of cardiac rehabilitation services are currently failing to meet important service performance indicators [7], a cost-effective method of support is needed to tie in with the current services provided. The PACES education programme combined with follow-up text-message support is low cost in application, designed to complement the current post-operative services available and could be easily translated into post-operative cardiac care should the intervention be successful.

As reported in the National Audit of Cardiac Rehabilitation Annual Statistical Report 2016 [7], the current dominant profile of individuals attending cardiac rehabilitation in the UK is male, White British, married and retired. The reason for non-completion of cardiac rehabilitation is still unclear, thus making it challenging to design an education programme which targets and overcomes specific barriers. In order to address this the development of the PACES programme encompassed a large element of PPI work, targeting as wide a population as possible and appealing to both those who did and did not take part in cardiac rehabilitation. This development process ensured that the programme provides added value to current cardiac care provision.

Structured education programmes underpinned by theory and delivered using a curriculum have been recognised by NICE and shown to be successful in the prevention and management of other long-term conditions such as type 1 diabetes mellitus (DAFNE; Dose Adjustment for Normal Eating [55]) and type 2 diabetes mellitus (DESMOND; Diabetes Education and Self-Management in On-going and Newly Diagnosed [56] and XPERT diabetes education courses [57]). The PACES structured education programme has been designed according to the Medical Research Council guidelines for developing and evaluating complex interventions in health-behaviour-change programmes whilst being established and refined in conjunction with extensive PPI work and through the use of existing infrastructure to meet the needs of patients in Leicestershire 12 to 48 months after a cardiac event [56]. A well-established training and monitoring plan has also been developed to support the PACES facilitators. This is to ensure that the PACES programme can be easily disseminated to the necessary health care professionals likely to deliver this programme to ensure the quality of delivery and ultimately consistency amongst facilitators.

The importance of managing CHD and preventing secondary events in at-risk individuals is recommended in national guidelines [5, 6]. Currently, patients’ long-term management plans should be a collaboration between the patient and primary and secondary care services [8], where structured physical activity, BMI and smoking are main focal points [6, 15]. It is also recognised that timely cardiac rehabilitation optimises patient outcomes and support thereafter is limited [9]. It is, therefore, important to assess the effectiveness and acceptability of lifestyle education focussing on self-managing CHD risk factors in secondary prevention. The current study will provide us with additional information to inform this research area.

### Trial status

Recruitment started on the 13 March 2017 and target recruitment is expected to be reached by the end of March 2018.

### Protocol version

The current protocol is Version 5; 16 August 2017. Two substantial amendments to the protocol have been approved. Substantial amendment 1 (before recruitment started) involved a change in the method of implementing randomisation from an online software system to an independent statistician developing the randomisation schedule. Substantial amendment 2 involved (1) widening the eligibility criteria from 3 years post cardiac event to 4 years post cardiac event and (2) permission to call non-responders to the postal invitation in order to confirm that they received the invitation and to ask if they have any questions about participation.

## Additional file

Additional file 1: Standard Protocol Items: Recommendations for Interventional Trials (SPIRIT) 2013 Checklist: recommended items to address in a clinical trial protocol and related documents. (DOC 120 kb)

## Abbreviations

BHF: British Heart Foundation
BLS: Basic life support
BMI: Body Mass Index
CHD: Coronary heart disease
CR: Cardiac rehabilitation
CVD: Cardiovascular disease
DAFNE: Dose Adjustment for Normal Eating
DESMOND: Diabetes Education and Self-Management in On-going and Newly Diagnosed
ENMO: Euclidean norm minus one
HADS: Hospital Anxiety and Depression Scale
HbA1c: Glycated haemoglobin
ILS: Immediate life support
ISWT: Incremental Shuttle Walk Test
MI: Myocardial infarction
MVPA: Moderate to vigorous physical activity
NICE: National Institute for Health and Care Excellence
NIHR: National Institute of Health Research
PACES: Physical Activity after Cardiac EventS
PPI: Patient and public involvement
RCT: Randomised controlled trial
RPAQ: Recent Physical Activity Questionnaire
RPD: Rating of perceived dyspnoea
RPE: Rating of perceived exertion
SAE: Serious adverse events
SOP: Standard operating procedure
